# The Quality of Recovery after Dexamethasone, Ondansetron, or Placebo Administration in Patients Undergoing Lower Limbs Orthopedic Surgery under Spinal Anesthesia Using Intrathecal Morphine. A Randomized Controlled Trial

**DOI:** 10.1155/2020/9265698

**Published:** 2020-05-20

**Authors:** Eduardo Toshiyuki Moro, Miguel Antônio Teixeira Ferreira, Renyer dos Santos Gonçalves, Roberta Costa Vargas, Samira Joverno Calil, Maria Alice Soranz, Joshua Bloomstone

**Affiliations:** ^1^Department of Surgery, School of Medical and Health Sciences, Pontifical Catholic University of São Paulo, Sorocaba, São Paulo, Brazil; ^2^School of Medical and Health Sciences, Pontifical Catholic University of São Paulo, São Paulo, Brazil; ^3^University of Arizona College of Medicine-Phoenix, Phoenix, AZ, USA; ^4^Division of Surgery and Interventional Sciences, University College London, London, UK; ^5^Envision Physician Services, Plantation, Florida, USA

## Abstract

Intrathecal morphine is widely and successfully used to prevent postoperative pain after orthopedic surgery, but it is frequently associated with side effects. The aim of this study was to evaluate the effect of dexamethasone or ondansetron when compared to placebo to reduce the occurrence of these undesirable effects and, consequently, to improve the quality of recovery based on patient's perspective. *Methods*. One hundred and thirty-five patients undergoing lower extremity orthopedic surgery under spinal anesthesia using bupivacaine and morphine were randomly assigned to receive IV dexamethasone, ondansetron, or saline. On the morning following surgery, a quality of recovery questionnaire (QoR-40) was completed. *Results*. No differences were detected in the global and dimensional QoR-40 scores following surgery; however, following postanesthesia care unit (PACU) discharge, pain scores were higher in patients receiving ondansetron compared with patients who received dexamethasone. *Conclusion*. Neither ondansetron nor dexamethasone improves the quality of recovery after lower limbs orthopedic surgery under spinal anesthesia using intrathecal morphine.

## 1. Introduction

Intrathecal morphine administration is recognized as one of the most efficacious approaches to preventing postoperative pain following lower extremity orthopedic surgery. However, for many patients who benefit from this approach, neuraxial morphine is also responsible for undesirable effects such as nausea, vomiting, pruritus, urinary retention, and respiratory depression [1, 2]. Of these, nausea, vomiting (60–80%) [3], and pruritus (20–100%) [4] are the most frequent. The administration of prophylactic therapies, such as dexamethasone [5] or ondansetron [6, 7], may reduce the occurrence of these complications. To this point, Szarvas et al. [8] evaluated the effect of single or combination therapy on the prevention of nausea, vomiting, and pruritus in patients undergoing orthopedic surgery under spinal anesthesia using bupivacaine and morphine. They concluded that ondansetron (8 mg) was more effective than dexamethasone (8 mg) in mitigating these side effects, and combination therapy was not better than ondansetron alone. It is important to state that, up to 0.7 mg of intrathecal morphine was used in this trial, which is considerably higher than in previous studies. In patients undergoing total hip arthroplasty under spinal anesthesia using morphine 0.1 mg, postoperative pain scores were similar to those in patients who received 0.2 mg. However, higher doses were associated with greater rates of side effects including hypotension and pruritus [9]. Recently, the global evaluation of the quality of postanesthetic recovery, from the patient's perspective, has become recognized as an important outcome measure in clinical trials comparing therapeutic alternatives. To this end, we have designed a prospective, randomized, double-blinded, and placebo-controlled study to evaluate, as a primary outcome, the quality of recovery, using the *Quality of Recovery Questionnaire* (QoR-40) [10], in patients undergoing lower extremity orthopedic surgery under spinal anesthesia using intrathecal morphine (0.1 mg) and receiving either ondansetron, dexamethasone, or placebo. We also evaluate postoperative pain scores; the incidence of nausea, vomiting, pruritus, and urinary retention; use of analgesics; and PACU length of stay.

## 2. Methods

### 2.1. Study Population

The Research Ethics Committee of the School of Medical and Health Sciences, Pontifical Catholic University of São Paulo (Sorocaba, São Paulo-Brazil), on 14 June 2016, CAAE 56610216.4.0000.5373 (Chairperson Prof. J.A. Costa), approved this randomized trial. The study was registered at NCT03035942, and written informed consent was obtained from each subject. From January 02, 2017, to January 31, 2018, patients undergoing lower limbs orthopedic surgery at Santa Lucinda hospital were recruited. The study included patients aged 18 to 60 years, with an ASA physical status of I or II, undergoing orthopedic surgery to treat fractures in one of the lower extremities. Exclusion criteria included (i) refusal to participate; (ii) lack of ability to communicate due to alterations in the level of consciousness or neurologic or psychiatric disease; (iii) presence of contraindication to spinal anesthesia or to any of the drugs used in the present study; (iv) alcohol or drug abuse; and (v) use of antiemetic agents or a history of surgery within 10 days of surgery. Reasons for exclusion following randomization included protocol violations and lost to follow-up. Subjects were randomly allocated into three groups according to a computer-generated table of random numbers (http://www.random.org): D (dexamethasone), O (ondansetron), and P (saline). For each patient included in the study, sequentially numbered opaque and sealed envelope containing group assignment was opened upon entry into the operating room. Study formulations were prepared by a nurse independent of the study. Normal saline (5 ml total volume) or dexamethasone 8 mg (made up to 5 ml with normal saline) or ondansetron (made up to 5 ml with normal saline) was drawn into each syringe. All care providers, researchers, and patients were blinded to group assignments.

### 2.2. Anesthetic Management

After informed consent was obtained, the baseline QoR-40 questionnaire was provided to subjects, and standard ASA monitors were applied. Before inducing spinal anesthesia, IV midazolam was titrated to achieve sedation (3 or 4 according to Ramsay scale) [11]. With patient in sitting position, a 26-gauge Quincke needle (B. Braun Melsungen S.A) was inserted into the L2-L3 or L3-L4 interspace. Anesthesia was established using a single injection of 0.5% hyperbaric bupivacaine (17.5 mg if weight >70 kg and/or expected duration >150 minutes or 15 mg if weight <70 kg) and preservative-free morphine 0.1 mg. Lactated Ringer's solution was used for fluid replacement therapy. In case of spinal anesthesia failure, a second attempt was allowed, or the technique was changed to general anesthesia, and the patient was excluded. Intraoperative sedation was based on titrated doses of IV midazolam (max 10 mg, including midazolam given before spinal anesthesia) and, if necessary, using propofol infusion to achieve Ramsay score level 4 in all patients in order to avoid different patients' experiences during the intraoperative period. We believe that these agents would not affect a patient's ability to respond to the questionnaire as it was applied 24 hours after the surgery. Data related to age, gender, physical status, and surgical duration were recorded. During PACU stay, clinical recovery variables such as pain score, analgesic use, occurrence of nausea, vomiting, pruritus, urinary retention, and PACU length of stay (time to Aldrete score ≥9) were assessed. Pain was rated by the subjects every 15 minutes based on a 0 to 10 pain numeric rating scale (NRS), where zero meant no pain and 10 meant the worst imaginable pain. IV morphine was administered every 10 minutes to maintain NRS <4 (1 mg for pain <7 and 2 mg for pain ≥7). Nausea (or vomiting) and pruritus were treated using IV dimenhydrinate (30 mg) and IV nalbuphine (5 mg), respectively. Multimodal analgesia consisting of IV ketoprofen 100 mg every 12 hours and IV dipyrone 30 mg·kg^−1^ every 6 hours was applied to all patients following PACU discharge. IV tramadol 100 mg (minimal interval of 8 hours) was administered when patients considered their pain control to be insufficient. Postoperative nausea (or vomiting) and pruritus were treated using IV dimenhydrinate (30 mg) and clorpheniramine (4 mg), respectively. The highest pain score (NRS); analgesic use; and the occurrence of nausea, vomiting, pruritus, and urinary retention were recorded.

### 2.3. Questionnaire

The QoR-40 questionnaire was completed by patients in the preoperative holding area and 24 hours after surgery. Data collection was performed by a blinded investigator. The QoR-40 assesses five dimensions of postoperative functional recovery: physical comfort, emotional status, physical independence, physiological support, and pain. The total score on the QoR-40 ranges from 40 (very poor quality of recovery) to 200 (excellent quality of recovery) [10].

### 2.4. Statistical Analysis

The primary outcome was the QoR-40 score 24 hours after surgery. Murphy et al. evaluated the quality of recovery in patients undergoing laparoscopic cholecystectomy with or without IV dexamethasone. The sample size estimated to achieve 80% power to detect a 17-point difference in the QoR-40 was 30 subjects per group [12]. A difference of 10 points represents a 15% improvement in the quality of recovery [10, 13]. Considering possible dropouts, one hundred and thirty-five subjects were finally randomized into three groups. The Shapiro–Wilk test was used to test the hypothesis of a normal distribution. Ordinal and continuous data that were not normally distributed are presented as median and range and were compared using the Kruskal–Wallis test. Dunn's multiple comparisons test was used to compare groups whenever a difference was detected. Multiple comparisons were tested at a 1.67% level (Bonferroni comparison). Statistical significance (*p* value) was assessed by means of a two-tailed test in all instances; values below 0.05 were considered statistically significant. Statistical analysis was performed using O IBM SPSS Statistics, version 22.

## 3. Results

**Figure 1:**
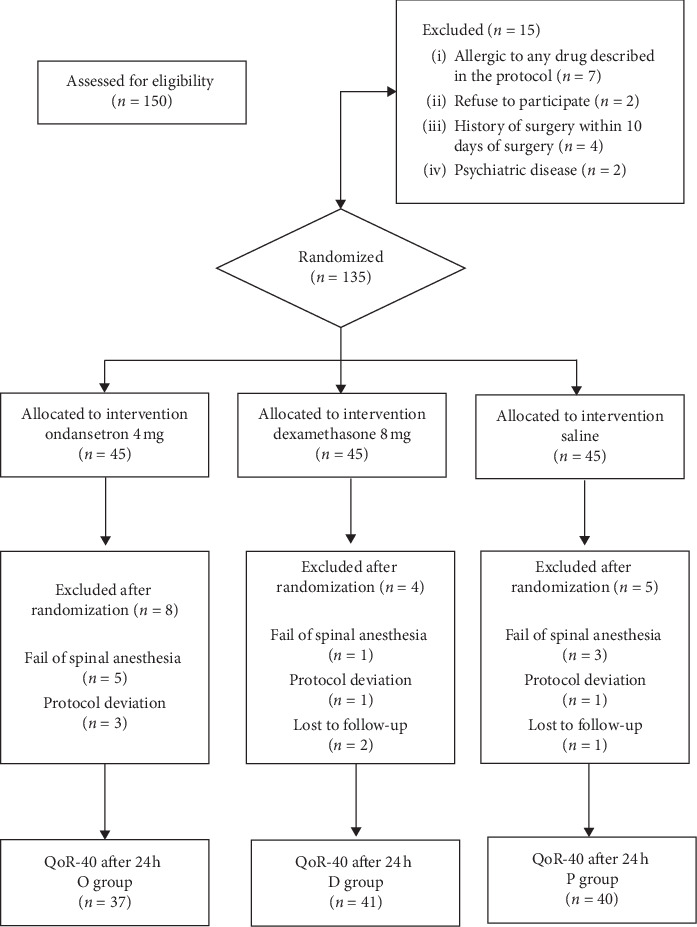
Flow study diagram.

One hundred and fifty patients were first assessed for eligibility. Fifteen subjects were excluded before randomization due to allergic to drugs included in the study (*n* = 7), refuse to participate (*n* = 2), a history of surgery within 10 days of surgery (*n* = 4), and previous diagnosis of psychiatric disease (*n* = 2). Thus, 135 patients were randomized and allocated into three groups. Later, seventeen subjects (D group = 4, P group = 5, and O group = 8) were excluded due to lost to follow-up (investigator-related), fail of spinal anesthesia, change of anesthesia technique, or protocol deviation (Figure 1).

Demographic data and surgery duration were similar between groups (Table 1).

The preoperative and postoperative global and dimensional QoR-40 scores were not different considering patients receiving ondansetron, dexamethasone, or saline (Table 2).

During PACU stay, recovery characteristics were assessed. No differences were detected in pain score; analgesic use; occurrence of nausea, vomiting, pruritus, or urinary retention; and time to achieve Aldrete score ≥9 (Table 3).

During the ward stay, patients were asked to rate the highest pain score (NRS) during the first 24 hours after surgery. These data were compared between groups. Pain scores were similar when ondansetron and dexamethasone groups were compared to the placebo group. However, subjects receiving ondansetron reported higher scores compared to those receiving dexamethasone (median difference = 5; 95% CI = 4 to 7; *p*=0.003) (Table 4).

The analgesic use and occurrence of nausea, vomiting, pruritus, and urinary retention were not different among groups during ward stay (Table 5).

## 4. Discussion

Despite the well-known analgesic benefits of intrathecal morphine, the occurrence of undesirable side effects is likely to impact the quality of recovery following anesthesia. Accordingly, pharmaceutical prophylaxis could improve both patient experience and satisfaction following anesthesia. In the current investigation, when compared to placebo, neither ondansetron nor dexamethasone improved the quality of recovery (QoR-40) in patients undergoing lower extremity orthopedic surgery under spinal anesthesia with bupivacaine and intrathecal morphine (0.1 mg). Multiple factors might influence a patient's perspective relative to the quality of postoperative recovery including, importantly, nausea and vomiting. In our study, 30% of patients experienced postsurgical nausea and emesis, and neither ondansetron nor dexamethasone decreased the occurrence of these complications compared to placebo, a result which clearly diverges from the published literature. Two systematic reviews demonstrate the efficacy of dexamethasone and ondansetron in reducing PONV in patients undergoing both cesarean delivery and abdominal hysterectomy under neuraxial anesthesia with intrathecal morphine [5, 14]. In patients undergoing orthopedic surgery under hyperbaric 0.5% bupivacaine spinal and intrathecal morphine, Szarvas et al. [8] assessed the antiemetic effect of dexamethasone (8 mg), ondansetron (8 mg), and the combination of dexamethasone (8 mg) and ondansetron (4 mg) and concluded that ondansetron alone was superior to single or combination therapy with dexamethasone. It is important to state that our study was properly powered to detect a difference in the QoR-40 score, but it may not have had sufficient power to detect differences in secondary outcomes such as nausea and vomiting. It is possible that a larger sample size might have detected some difference. The analgesic benefit of a single perioperative dose of dexamethasone is well known, but in the present study, this effect was not observed. An interesting finding in our study was that patients who received ondansetron had higher pain scores during the first 24 hours after surgery than patients who received dexamethasone (*p*=0.003). Though we have no explanation as to why the ondansetron group experienced more pain than the dexamethasone group, it is possible that the 5-HT(3) spinal receptor plays a role in acute pain modulation in humans. Indeed, Arcioni et al. [15] have shown that ondansetron inhibits the analgesic effects of tramadol, used as analgesic rescue in our study, which if true, may substantially alter the routine perioperative use of 5-HT(3) receptor antagonists. Opioid-induced pruritus, an unpleasant and frequent postoperative adverse occurrence, is surely a cause of poor quality of recovery. Unfortunately, 30%–60% of patients experience pruritis following orthopedic surgery, a problem for which neither the mechanism nor prevention or treatment is clearly known [16, 17]. Different pharmacological therapies have been proposed including propofol, antihistamines, 5-HT(3) receptor antagonists, and naloxone [18]. In patients undergoing cesarean section under spinal anesthesia with intrathecal morphine, ondansetron administration decreased the intensity of pruritus and the need for treatment, but not its incidence [18]. Swaro et al. [19] assessed the antipruritic effects of dexamethasone, palonosetron, and the combination of both. The authors concluded that palonosetron, with or without dexamethasone, was more effective when compared to dexamethasone alone. In the current investigation, 45% of our patients experienced postoperative pruritus, and neither dexamethasone nor ondansetron decreased the incidence of this unpleasant occurrence. Our study presents several limitations. First, this is a single-center study, and thus our results may not be generalizable. Second, this study was powered to detect a difference in the primary outcome, but it may not have had sufficient power to detect differences in secondary outcomes. Third, this investigation did not assess the quality of recovery beyond 24 hours and thus offers no insights into the potential benefits of ondansetron and/or dexamethasone beyond the first postoperative day. Fourth, we do not know whether higher doses of dexamethasone (>8 mg) and ondansetron (>4 mg) would provide better quality of recovery. Two systematic reviews concluded that a dose more than 0.1 mg/kg is effective in reducing postoperative pain [20] and PONV [21], and the recommended dose of ondansetron based on a recent Consensus Guidelines for the Management of Postoperative Nausea and Vomiting is 4 mg [22]. Further studies will be required to ascertain how these medications affect the quality of longer-term surgical recovery.

In conclusion, neither ondansetron nor dexamethasone improves the quality of recovery as assessed by the QoR-40 questionnaire in patients undergoing lower extremity orthopedic surgery under spinal anesthesia using 0.5% hyperbaric bupivacaine and intrathecal morphine.

## Figures and Tables

**Table 1:** Patients' characteristics and operative data. Group O = ondansetron 4 mg, Group D = dexamethasone 8 mg, and Group P = saline.

	Group O (*n* = 37)	Group D (*n* = 41)	Group P (*n* = 40)
Age (years)	34 (24–47)	40 (26–54)	35 (25–49)
*ASA*			
I	23 (62.2%)	21 (51.22%)	24 (60.0%)
II	14 (37.8%)	20 (48.8%)	16 (40.0%)
*Gender*			
Female	13 (35.1%)	11 (26.8%)	11 (27.5%)
Male	24 (64.9%)	30 (73.2%)	29 (72.5%)
Body mass index (kg/m^−2^)	25 (24–28)	26 (23.9–28.0)	26.75 (24–29.2)
Surgical duration (min)	100 (85–120)	120 (90–150)	100 (75–120)

Data are presented as median (interquartile range) or *n* (%). Data were analyzed using Kruskal–Wallis test.

**Table 2:** Dimensions of the quality of recovery 40 (QoR-40) questionnaire by study groups preoperatively and at 24 hours after surgery (POD1). Group O = ondansetron 4 mg, Group D = dexamethasone 8 mg, and Group P = saline.

	Group O (*n* = 37)	Group D (*n* = 41)	Group P (*n* = 40)	*p* value
*Preoperative*				
Emotional status	44 (40–45)	44 (41–45)	42 (38–45)	0.26
Physical comfort	59 (56–60)	59 (56–60)	58 (57–60)	0.25
Psychological support	40 (38–40)	40 (40–40)	40 (39–40)	0.85
Physical independence	20 (16–20)	20 (17–20)	20 (17–20)	0.07
Pain	35 (33–35)	35 (33–35)	35 (32–35)	0.76
Total QoR-40	194 (185–198)	195 (185–198)	192 (184–196)	0.60

*POD1*				
Emotional status	42 (39–44)	44 (41–45)	43 (38–45)	0.36
Physical comfort	56 (52–58)	58 (55–60)	57 (51–59)	0.20
Psychological support	40 (38–40)	40 (38–40)	40 (36–40)	0.05
Physical independence	18 (15–20)	16 (13–20)	16 (15–18)	0.57
Pain	34 (31–35)	35 (32–35)	34 (32–35)	0.45
Total QoR-40	184 (179–195)	187 (181–196)	185 (176–196)	0.14

Data are presented as median (interquartile range). Data were analyzed using Kruskal–Wallis test.

**Table 3:** Postanesthesia care unit parameters. Group O = ondansetron 4 mg, Group D = dexamethasone 8 mg, and Group P = saline.

	Group O (*n* = 37)	Group D (*n* = 41)	Group P (*n* = 40)	*p* value
Pain score (NRS)	0 (0–0)	0 (0–0)	0 (0–0)	0.99^a^
Morphine consumption (mg)	0 (0–0)	0 (0–0)	0 (0–0)	0.59^a^
PONV	8.1%	21.9%	22.5%	0.19^b^
Pruritus	10.8%	17.1%	20.0%	0.57^b^
Time to achieve Aldrete score ≥9 (min)	75 (60–100)	70 (60–100)	65 (60–93)	0.95^a^
Urinary retention	21.6%	9.8%	5.0%	0.08^c^

Data are presented as median (interquartile range) or *n* (%). Pain score results represent the average value of NRS. Continuous and ordinal data were analyzed using Kruskal–Wallis test^a^. Categorical data were compared with Chi-square test^b^ or Fisher's exact test^c^.

**Table 4:** Pain scores during ward staying. Comparison between groups dexamethasone and ondansetron (D × O), groups dexamethasone and saline (D × P), and groups ondansetron and saline (O × P).

	Groups D × O	Groups D × P	Groups O × P
Pain score (ward)	0.003	0.39	0.20

Data are presented as *p* value. Data were analyzed using Dunn's Multiple Comparisons Test. Multiple comparisons were tested at a 1.67% level (Bonferroni comparison).

**Table 5:** Ward parameters.

	Group O (*n* = 37)	Group D (*n* = 41)	Group P (*n* = 40)	*p* value
Pain score (NRS)	5 (3–7)	0 (0–5)	2 (0–6)	0.004^a^
Tramadol consumption	100 (0–100)	100 (0–200)	100 (0–200)	0.394^a^
Nausea or vomiting	29.7%	24.4%	37.5%	0.44^b^
Pruritus	51.3%	46.3%	40.0%	0.62^b^
Urinary retention	24.3%	34.1%	17.5%	0.22^b^

Data are presented as median (25%–75% interquartile range) or *n* (%). Continuous and ordinal data were analyzed using Kruskal–Wallis test^a^. Categorical data were compared with Chi-square test^b^.

## Data Availability

The datasets generated and analyzed during the current study are available from the corresponding author upon request.
